# Cellular Defense Enzyme Drives Exceptionally High Rate of Mutation in HIV

**DOI:** 10.1371/journal.pbio.1002252

**Published:** 2015-09-16

**Authors:** Richard Robinson

**Affiliations:** Freelance Science Writer, Sherborn, Massachusetts, United States of America

## Abstract

HIV-1 is already known to have an extremely fast mutation rate, but a new study shows it to be more than two orders of magnitude higher than previously believed, and that this is largely due to host cytidine deaminases. Read the Research Article.

Mutation is the raw material of evolution and a necessary precondition for adaptation to a hostile environment. On the other hand, mutation changes what works, and rarely for the better, a truth equally relevant for a virus as for a human. In a new study in PLOS Biology, José Cuevas, Ron Geller, Rafael Sanjuán, and colleagues measured the mutation rate of HIV-1, the virus that causes AIDS, in patient´s cells (Fig 1). They show that the HIV-1 mutation rate is far higher than previously thought, due mainly to the efforts of a cellular enzyme. The high rate of mutation caused by the enzyme may protect the host from virulence but may also promote evolution of HIV to escape that protection.

**Fig 1:**
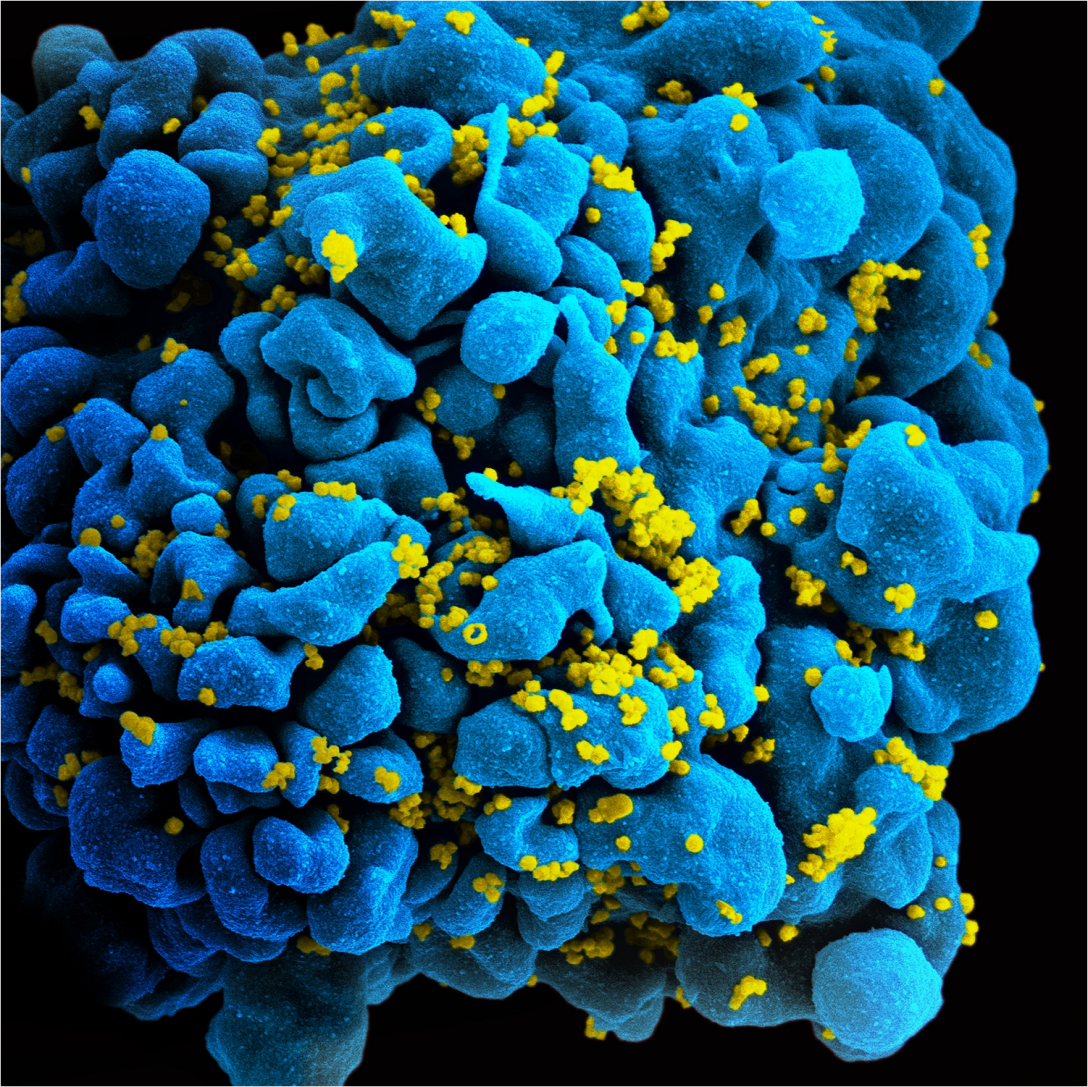
HIV-1 infected cell. The image shows a microphotography of an infected cell obtained with a scanning electron microscope. The cellular surface and budding viruses have been colored. *Image credit*: *NIAID*, *Flickr*.

The authors compared the mutation rate in HIV isolated from its cellular host to that of virus particles released into blood plasma. In plasma, they found an average of 9.3 mutations per 10,000 bases (the HIV genome comprises 9,181 base pairs). But inside infected cells, the rate was more than 40 times higher, at 410 per 10,000 bases, the highest rate of mutation in any known virus. Most of the mistakes were lethal, preventing the virus from exiting the cell, accounting for the large discrepancy between mutation rates measured within and outside the cell.

What led to the high rate of error? HIV has no ability to edit the copying mistakes made by its own reverse transcriptase, so some fraction of the mutations were likely viral in origin. Another source of mutation was the group of cellular enzymes from the A3 family, which cause G-to-A substitutions in viral RNA. A3 enzymes are packaged into the virion and travel with it, inducing mutations when the virus enters a new host cell.

To determine the relative contributions of viral copying mistakes and A3-induced mutations, the authors looked at the sequence context of the lethal mutations. There are multiple members of the A3 family, each with its preferred sequence targets. Among the most prolific member in HIV-infected cells is A3G, which converts tryptophan TGG codons to either TAG, TGA, or TAA, depending on the bases surrounding the codon. Errors made by reverse transcriptase, on the other hand, do not show this sequence context preference. Thus, by analyzing the sequence surrounding the mutations, the authors could assign each to either reverse transcriptase or one of the A3 enzymes. Their analysis showed that only 2% of errors could be attributed to reverse transcriptase; the rest were the work of one of the A3 enzymes.

The data here were obtained from 11 individuals infected with HIV who were not yet receiving treatment. The authors found that the mutation rate in rapid progressors (those whose T cell counts declined the fastest) was lower than that in normal progressors, supporting the idea that A3-directed mutation is a defense mechanism against HIV. Further support came from one patient carrying a mutation in a critical region of a gene called virulence factor, whose protein product attaches to A3 and induces the cellular quality control machinery to degrade it. The mutation in this patient is predicted to reduce virulence factor’s ability to degrade A3. The overall mutation rate in this patient was four times that of the average of the other patients, suggesting that the virus’ inability to disable A3 led to the increase in the mutation rate.

Yet while a high mutation rate seems to be helpful in defending against infection, the authors found some evidence that a low mutation rate may not be better than none at all. Rapid progressors in this study had higher rates of low-level A3 editing (i.e., editing that introduced few mutations per genome) than normal progressors, and the rate of low-level editing (which was nonetheless higher than reverse transcriptase alone) correlated with a higher viral load. Together, these results suggest that the increase in genetic diversity from a small number of mutations may promote adaptation rather than fight infection.

More work in larger numbers of patients will be needed to confirm these results, but the implications are important for understanding, and treating, HIV infection. Mutation may be lethal, but it also leads to development of drug resistance and thwarts attempts at vaccination. Understanding how mutation is governed in HIV may lead to better strategies for promoting its antiviral benefits and preventing its liabilities.
